# A 4-Week Model of House Dust Mite (HDM) Induced Allergic Airways Inflammation with Airway Remodeling

**DOI:** 10.1038/s41598-018-24574-x

**Published:** 2018-05-02

**Authors:** L. N. Woo, W. Y. Guo, X. Wang, A. Young, S. Salehi, A. Hin, Y. Zhang, J. A. Scott, C. W. Chow

**Affiliations:** 1 Division of Respirology and Multi-Organ Transplant Programme, University Health Network, Faculty of Medicine, Toronto, Canada; 2 Division of Occupational and Environmental Health, Dalla Lana School of Public Health, University of Toronto, Toronto, Canada

## Abstract

Animal models of allergic airways inflammation are useful tools in studying the pathogenesis of asthma and potential therapeutic interventions. The different allergic airways inflammation models available to date employ varying doses, frequency, duration and types of allergen, which lead to the development of different features of asthma; showing varying degrees of airways inflammation and hyper-responsiveness (AHR) and airways remodeling. Models that also exhibit airway remodeling, a key feature of asthma, in addition to AHR and airway inflammation typically require 5–12 weeks to develop. In this report, we describe a 4-week mouse model of house dust mite (HDM)-induced allergic airways inflammation, and compare the phenotypic features of two different doses of HDM exposures (10 µg and 25 µg) for 5 days/week with a well-characterized 8-week chronic HDM model. We found that 4 weeks of intranasal HDM (25 µg in 35 µl saline; 5 days/week) resulted in AHR, airway inflammation and airway remodeling that were comparable to the 8-week model. We conclude that this new 4-week HDM model is another useful tool in studies of human asthma that offers advantages of shorter duration for development and decreased costs when compared to other models that require longer durations of exposure (5–12 weeks) to develop.

## Introduction

Asthma is a chronic inflammatory airway disease that affects 235 million people worldwide, and is associated with a significant health and economic burden^1,2^. The incidence of asthma continues to increase, despite the availability of effective bronchodilators and anti-inflammatory therapies^3^. It is increasingly recognized that asthma is a heterogeneous disease that can be clustered by different features, such as disease severity, predominant inflammatory phenotype (i.e., atopic, non-atopic, eosinophilic and/or neutrophilic), age of onset and response to corticosteroid therapy^1,4,5^. No therapy to date has been shown to reverse airway remodeling^6,7^, a key feature of asthma that can occur prior to the onset of symptoms^8,9^. Thus, the presence of airway remodeling is essential for the utility of a comprehensive animal model of asthma.

Multiple mouse models of allergic airways inflammation have been established to investigate the different features of asthma to date^10,11^. Each model varies with respect to the type, species, duration and dose of allergens used to induce the allergic airways, and include house dust mite (HDM), ovalbumin, molds and cockroach antigen^12–15^. The choice of models employed for experimental evaluation is often determined based on the specific aspects being examined in the development of airways inflammation, allergic responses and/or asthma. HDM exposures are very common in the home and built environment, and has become an increasingly common and clinically-relevant allergen in the experimental setting; inducing allergic responses in 85% of asthmatic individuals^16^. Acute models of allergic airways inflammation, typically induced in less than 3 weeks, often manifest the features of AHR and airways inflammation, but not remodeling^11,12,17,18^. Although observations of some aspects of airway remodeling such goblet cell hyperplasia have been reported in a 2-week model^17^, airway remodeling with collagen deposition does not typically occur until allergen exposure is more prolonged up to 5–12 weeks, which requires a significant investment in time and resources^17–25^. Thus, the development of an allergen exposure protocol that induces AHR, airways inflammation and airway remodeling with collagen deposition in the shortest duration of time would improve the selection of tools available to researchers for such studies.

In this paper, we describe two 4-week models of HDM-induced allergic airways inflammation using a lower (10 µg) and higher (25 µg) dose of *Dermatophagoides pteronyssinus*, and compare the phenotypic features to 2- and 8-week HDM models^22^. We found that 4 weeks of intranasal exposure to 25 µg HDM daily during the week, resulted in AHR, airways inflammation and remodeling with collagen deposition, which were comparable to the 8-week HDM-sensitization and -challenge model. We propose that this 4-week model is a suitable model of chronic allergic airways remodeling that offers significant advantage over the 8-week model due to the economy of time and resource utilization.

## Methods

### Animals

The animal protocols were approved by the University Health Network Animal Care Committee and conducted in accordance with the guidelines of the Canadian Council on Animal Care (CCAC). Female BALB/c mice (8–10 weeks old, Jackson Laboratories, Bar Harbor, ME, USA) were housed under pathogen-free conditions and a 12:12 hour light: dark cycle, and acclimatized for one week before the start of the experiments. The health status of the animals was monitored daily. None of the animals died or became ill prior to the study end-point.

### Development of allergic airways inflammation

**Figure 1:**
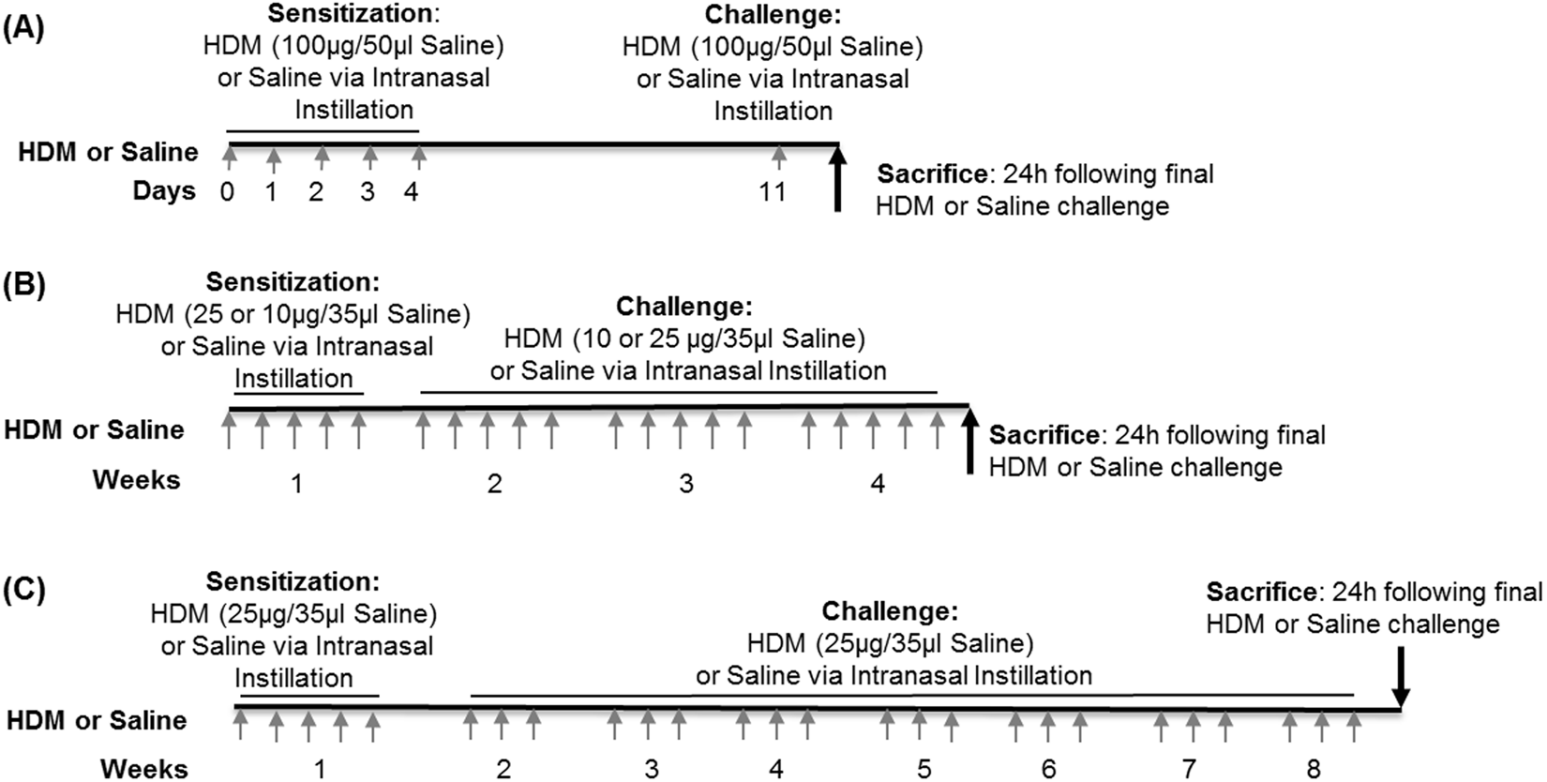
Timelines for allergen exposure in the 3 different HDM models. (**A**) Acute 2-week model of HDM-induced airways inflammation. Mice were sensitized by intranasal instillation of 100 µg HDM in 50 µl saline or 50 µl saline alone (Control) on days 0–4 and then challenged with 100 µg HDM on day 11. (**B**) Chronic 4-week model of HDM-induced airways inflammation. Mice were sensitized to 10 µg or 25 µg HDM in 35 µl saline or 35 µl saline alone by intranasal instillation for 5 days/week for 4 weeks. (**C**) Chronic 8-week chronic model of HDM-induced airways inflammation. Mice were sensitized by intranasal instillation of 25 µg HDM in 35 µl saline or 35 µl saline for 5 days a week in week 1, and every Monday, Wednesday and Friday in weeks 2–8.

HDM extract (Greer Laboratories, Lenoir, NC) was re-suspended in normal saline and introduced by intranasal instillation, with the mice lightly anesthetized with 5% isoflurane, using a 20–200 µL pipette tip (Fig. 1). The acute HDM protocol used 100 µg HDM (*D. pteronyssinus* or *D. farinae*) in 50 µL saline on days 0–4 and day 11. Saline-exposed control mice received 50 µL sterile saline. The 4-week models used 10 µg or 25 µg *D. pteronyssinus* in 35 µL of saline. Mice were sensitized for 5 consecutive days weekly (from Monday to Fridays) for 4 weeks. The mice in the 8-week model were sensitized to 25 µg *D. pteronyssinus* for 5 consecutive days on week 1, followed by every other weekday (Monday, Wednesday and Friday) from weeks 2 to 8. Saline-exposed control mice in the 2-week, 4-week and 8-week models were handled in a similar fashion but were only treated with sterile saline. Evaluation of the endpoint metrics occurred 24 hours after the last HDM or saline exposure.

### Assessment of lung function and methacholine responsiveness

Mice were anesthetized with ketamine (50 mg/kg i.p., Wyeth Animal Health, Guelph, ON, Canada) and xylazine (10 mg/kg i.p., Bayern Inc., Toronto, ON, Canada), and intubated intratracheally, for measurement of *in vivo* respiratory mechanics using the ventilator-based Flexi-Vent® system (SciReq Inc., Montreal, QC, Canada). Rocuronium (1.8–4.8 mg/kg i.p., Sandoz Canada Inc., Boucherville, QC, Canada) was administered prior to pulmonary function testing to prevent respiratory drive artifacts. Baseline measurements of respiratory mechanics (including quasistatic compliance; C_st_) were assessed prior to challenge with increasing concentrations of nebulized methacholine (MCh; Sigma-Aldrich, Mississauga ON), and determination of total respiratory resistance (R_rs_) and central airways Newtonian resistance (R_N_), as described previously^21,22,26^.

### Collection of Tissue

After completion of the pulmonary function testing, mice were euthanized with an overdose of ketamine/xylazine, and blood was collected via cardiac puncture^21,22^. In a subset of mice, bronchoalveolar lavage (BAL) was performed, and lungs were harvested and placed in RNAlater® (Life Technologies, Burlington, ON, Canada) for storage at −20 °C, or inflation-fixed to 25 cm H_2_O in 4% paraformaldehyde for subsequent histologic analysis^27^. BAL fluid was centrifuged (400 RPM, 10 min) to obtain cell-free supernatants and stored at −80 °C; the cell pellet was re-suspended in 500 ml phosphate buffered saline (PBS) for total leukocyte counts using a hemocytometer^21–23^. Differential leukocyte counts were performed using a StatSpin Cytofuge 2 (Beckman Coulter Inc, CA, USA), with 70 μl of the resuspended pellets stained using the HEMA3 stain set (Fisher Scientific, PA, USA).

### Quantification of HDM-Specific IgE in Serum

Serum HDM-specific IgE was assessed using the antigen-capture ELISA method^22^. Briefly, 96 well plates (NUNC MaxiSorp1 plates, Sigma Aldrich Co., Mississauga, ON) were coated with 5 µg HDM in 100 µl coating buffer and incubated overnight at 4 °C. Plates were blocked with 200 µl/well of assay diluent. Serum samples were precleared by overnight incubation with Protein G Sepharose beads (GE Healthcare) (1:1) at 4 °C^17^, and washed with PBS. A 50 µl aliquot of undiluted serum was added to each well and incubated at 4 °C overnight. The wells were washed with PBS; 100 µl of biotin-anti-mouse IgE (BioLegend, CA, USA) was added and incubated for 1 hr. followed by a 30 min incubation with avidin-horse radish peroxidase (Biolegend). TMB substrate solution (100 µl) was added to each well and incubated in the dark for 30 minutes. The reaction was stopped with 2 N H_2_SO_4_. Optical densities were read at 450 nm with reference at 570 nm using the Titertek Multiskan Ascent spectrophotometer (Titertek Instruments Inc., Huntsville, AL, USA). Data was normalized to the respective saline-exposed controls.

### Histological analysis

Fixed lung tissue was dehydrated using ethanol and xylene and embedded in paraffin (Electron Microscopy Science, Hatfield, PA, USA). Lung sectioning, subsequent staining with hematoxylin and eosin (H&E), Periodic acid-Schiff (PAS) and Masson’s Trichrome, and slide scanning (40X) were conducted at the Toronto Centre for Phenogenomics (Toronto, ON).

### Quantification of total collagen content

The total lung collagen content was assessed using the Quickzyme Total Collagen Assay Kit (Quickzyme Biosciences Inc, Leiden, Netherlands) according to the manufacturer’s instructions^22^. Briefly, 50 mg lung tissue was hydrolyzed in 6 M HCl at 95 °C for 20 h and diluted 15- and 20-fold for the 4-week and 8-week HDM models, respectively. Aliquots of tissue hydrolysate (35 μl) were incubated on a microtiter plate with 75 μl assay buffer for 20 min at room temperature. Seventy-five μl of detection reagents A and B (2:3 *v/v*) was added and the plate was incubated at 60 °C for 60 min. The plate was then cooled to room temperature and the optical density (O.D.) read at 570 nm. Standard curves to the collagen standard were prepared via serial dilutions. The collagen content was normalized to the weight of the lung to standardize the total collagen.

### Gene expression profiling

Total RNA was extracted using RNAeasy Mini Kit (Qiagen Inc, ON, Canada). The SYBR green Real Time Quantitative RT-PCR kit (Roche Bioscience) was used for qPCR using the CFX384 Touch Real-Time PCR Detection System (BioRad, ON, Canada). Primer sets are presented in Supplemental Table 1.

### Statistical analysis

Data are expressed as the mean ± SEM. Binary comparisons were made using Student *t* test or Mann-Whitney test, as appropriate. Multiple group comparisons were made using one-way ANOVA, with post hoc comparison via Tukey’s test, or Kruskal-Wallis test with Dunn’s post hoc test, as appropriate. Two-way ANOVA was conducted to compare individual dose differences between the MCh dose-response relationships. Statistical analyses were conducted using GraphPad Prism (version 7.0; GraphPad Software, La Jolla, CA).

## Results

### HDM Sensitization and Challenge Induces Airways Hyperresponsiveness in All Models

**Figure 2:**
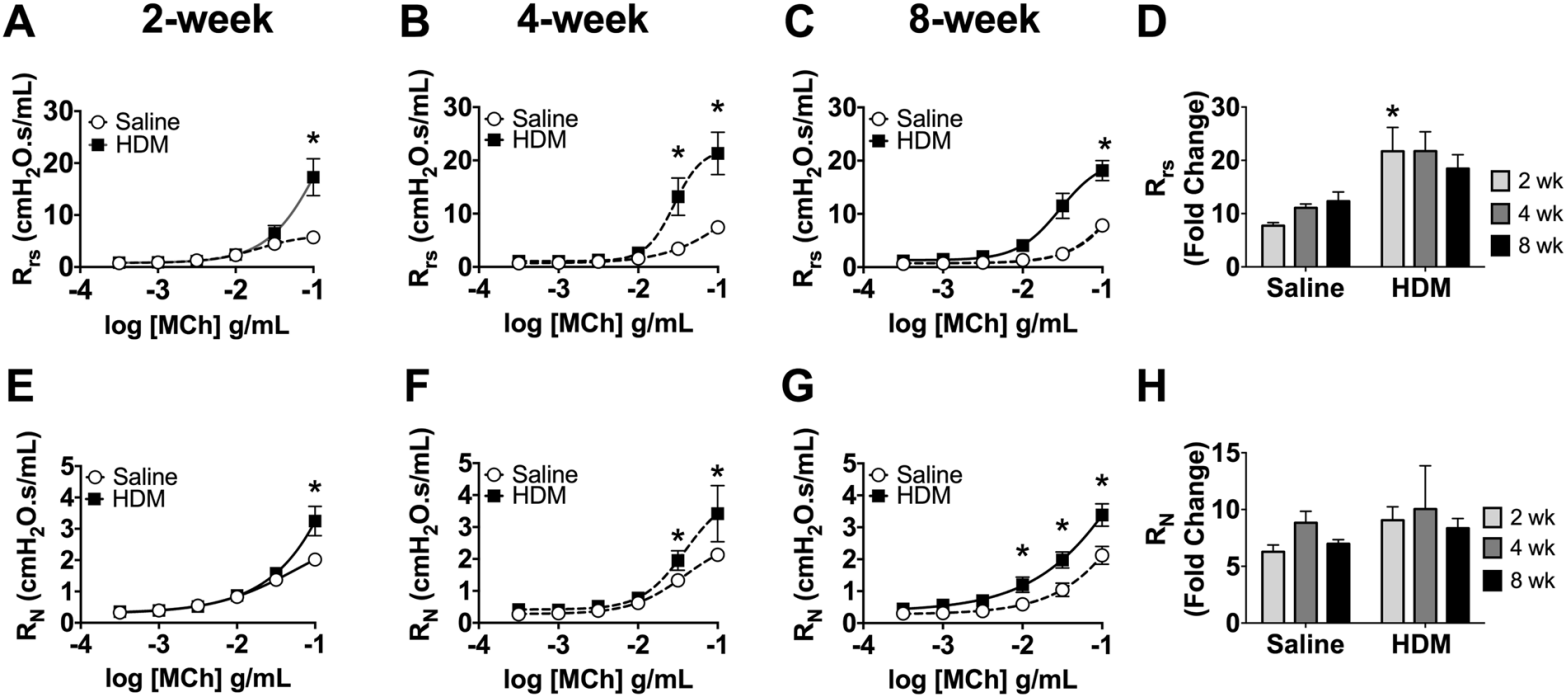
Enhanced methacholine responsiveness occurs in the 2, 4 and 8-Week *D. pteronyssinus* HDM models. Lung function assessment demonstrated that all *D. pteronyssinus -*exposed mice exhibited significant increases in total respiratory resistance, R_rs,_ and central airways resistance R_N_ to MCh relative to saline controls (**A**–**C**, **E**–**G**, *p < 0.05, n = 7–10/group). The increases in R_rs_ max (**D**) and R_N_ max (**H**) were similar in all *D. pteronyssinus* HDM groups compared to saline controls (*p < 0.05, n = 7–10/group).

Mice sensitized and challenged with *D. pteronyssinus* (HDM) for up to 8 weeks developed enhanced methacholine (MCh) responsiveness of the total respiratory system (R_rs_) and central airways Newtonian resistance (R_N_) in all models compared to saline-exposed controls (Fig. 2; *p < 0.05 to Saline, n = 7–10/group). Interestingly, mice sensitized and challenged with *D. farinae* did not develop AHR in the two-week model (Supplemental Fig. 2). The 2-week and 4-week 25 µg HDM models exhibited similar degrees of AHR to the previously established 8-week model with respect to both R_rs_, and R_N_.

### Development of the Allergic Response

**Figure 3:**
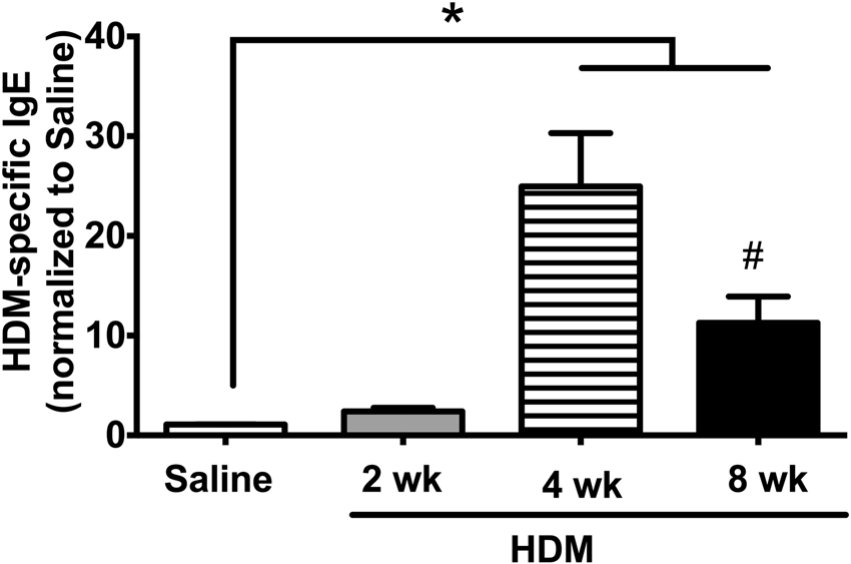
Serum HDM-specific IgE levels required more than 2 weeks of HDM sensitization to develop. Serum HDM-specific IgE was significantly increased in the 4-week (25 µg) and 8-week HDM models, with the highest concentrations in the 4-week HDM mice (*p < 0.05 vs Saline control; ^#^p < 0.05, 4-week HDM vs 8-week HDM; n = 6/group).

Serum HDM-specific IgE levels were elevated significantly compared with saline controls in the 4-week 25 µg and 8-week models (Fig. 3, *p < 0.05, Saline vs. HDM, n = 5–13/group), with the highest level observed in the 4-week 25 μg model. While we did observe a 2.4-fold increase in HDM-specific IgE in our 2-week model, this was not significant by one-way ANOVA.

### Airways Inflammation

**Figure 4:**
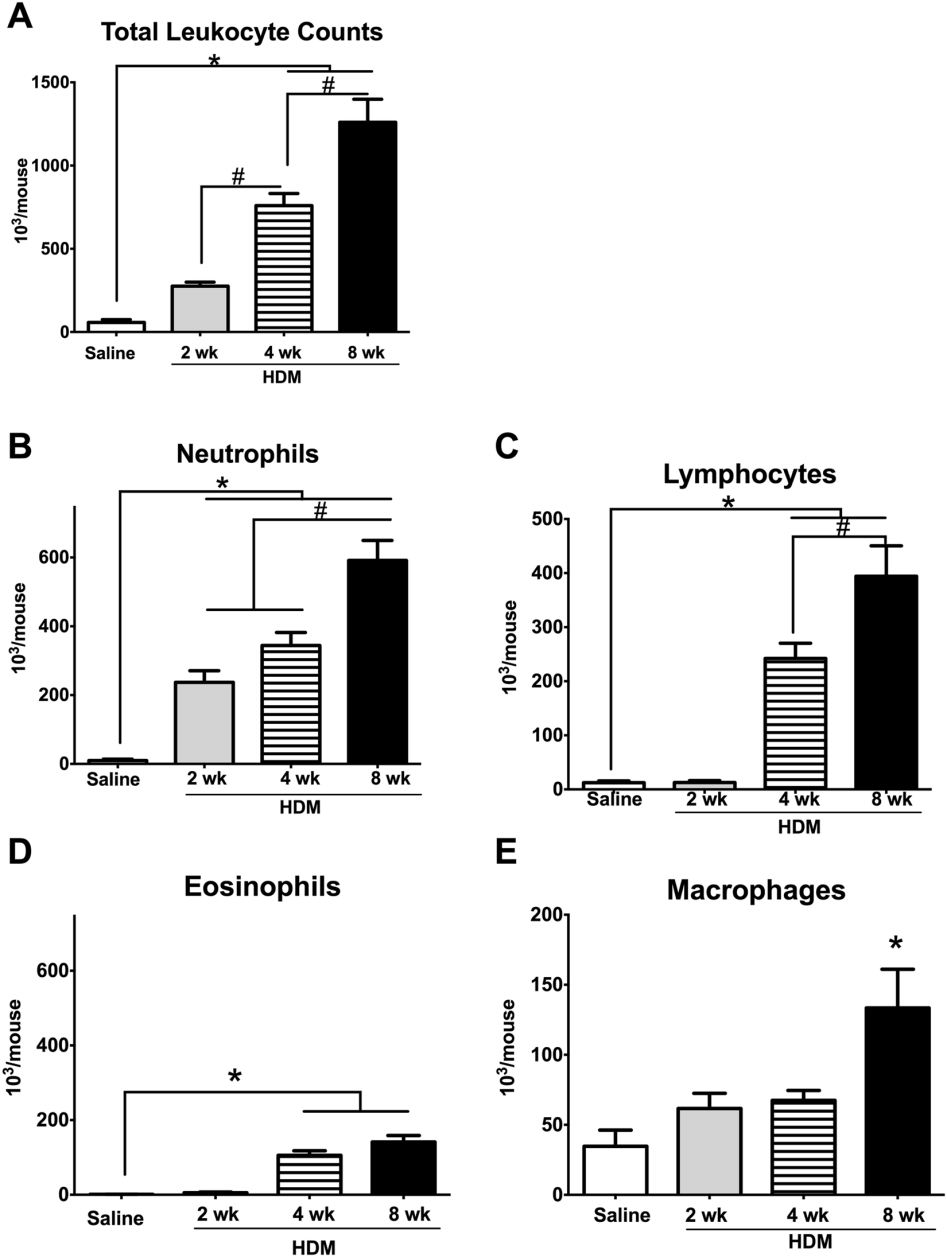
Increasing duration of HDM sensitization corresponds to increasing leukocyte recruitment to the airways. Total leukocyte counts in the BAL revealed a progressive increase in leucocyte recruitment with longer HDM exposures (**A**); *p < 0.05 vs Saline control, ^#^p < 0.05 within HDM groups, n = 7/group). Differential leukocyte counts revealed that the leukocytosis was primarily due to increases in neutrophils (**B**), lymphocytes (**C**) and eosinophils (**D**), while BALF macrophages were increased only in 8-week HDM mice. (**E**) *p < 0.05 vs Saline control, ^#^p < 0.05 within HDM groups, n = 7/group.

The BAL fluid total leukocyte counts showed progressive and significant increases over time (Fig. 4A, *p < 0.05, HDM vs. Saline, n = 5–7/group). Among all models, the 8-week HDM model exhibited the most robust cellular recruitment to the airways (Fig. 4A–E, *p < 0.05, HDM vs. Saline, n = 5–7/group). Differential counts revealed increases in the absolute cell counts of neutrophils, eosinophils and lymphocytes (Fig. 4B–D *p < 0.05). The increases in the eosinophils in the 4-week 25 µg and 8-week HDM models were consistent with the increases in the serum HDM-specific IgE. However, the relative increases in the proportions of eosinophils were significantly greater compared with the increases in neutrophils or lymphocytes. The relative increases in eosinophils in the HDM mice relative to Saline controls were 5.6-, 10.0- and 8.0-fold in the 2-, 4- and 8-week HDM mice, compared with 3.8-, 3.3- and 3.4-fold at 2-, 4- and 8-weeks, respectively, for the neutrophils, and 0.8-, 1.3- and 1.2-fold at 2-, 4- and 8-weeks, respectively for lymphocytes.

**Figure 5:**
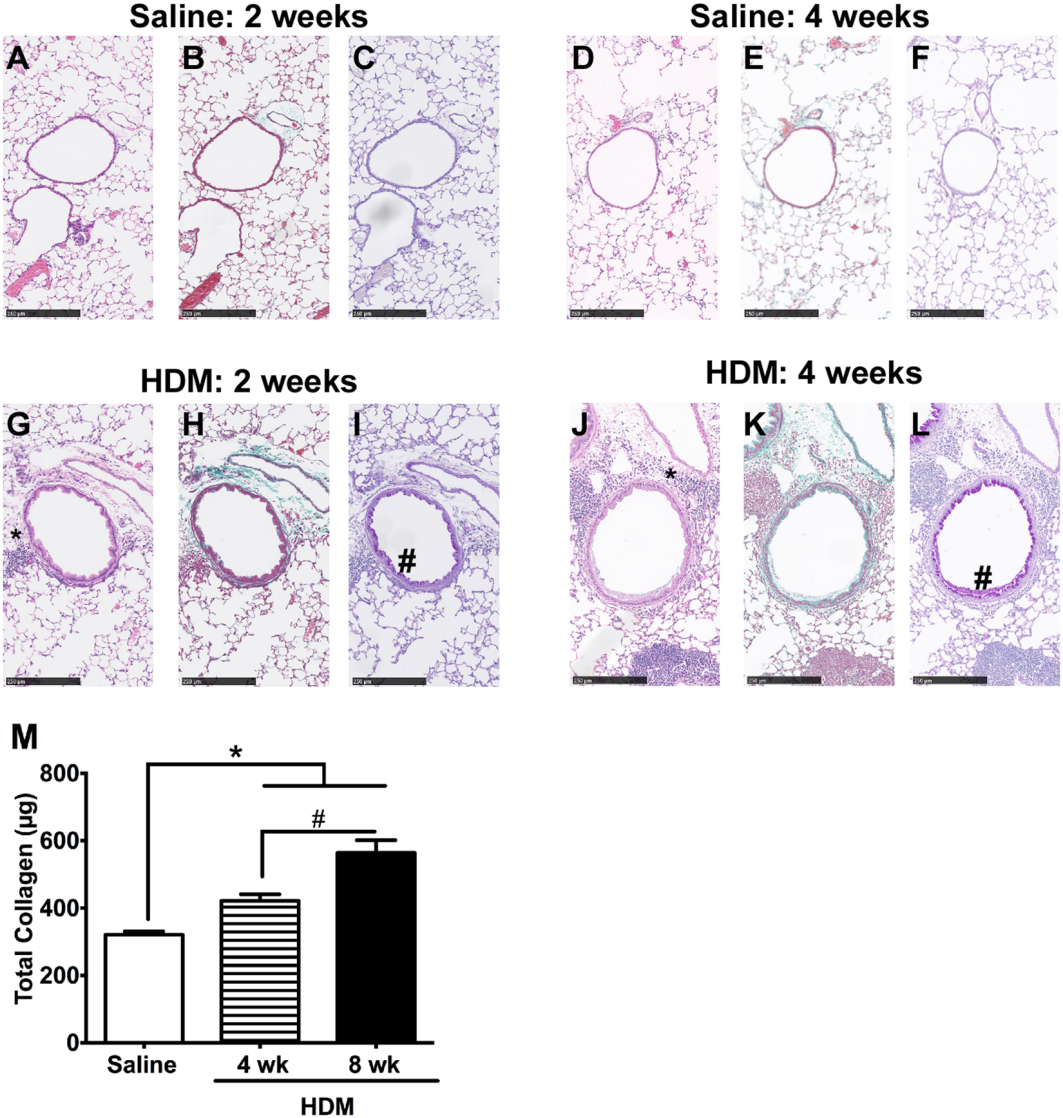
Airway remodeling with collagen deposition is observed in the 4-week HDM mice. Hematoxylin and eosin (**A**,**D**,**G**,**J**). Trichrome Mason staining (**B**,**E**,**H**,**K**) of paraffin embedded lung slices showed peribronchial inflammation infiltrates (*) in the 2-week (**G**) and 4-week (25 µg) HDM mice (**J**), while subepithelial collagen deposition (stained green) was only detected in the 4-week model (**K**). Periodic acid-Schiff staining (**C**,**F**,**I**,**L**) revealed goblet cell hyperplasia (#) in both the 2-week (100 µg HDM) (**I**) and 4-week (25 µg HDM) models (**L**). (Representative of 4 animals/group; size bar = 250 µm). Total collagen content (**M**) was increased in the 4- and 8-week HDM lungs, and was the highest at 8 weeks (p < 0.05 vs. Saline control, ^#^p < 0.05 between the 4- and 8-week HDM groups, n = 6/group).

Histological evidence of peribronchial inflammation was also observed in the HDM-exposed mice, and was more marked in the 4-week 25 µg model compared with the 2-week model (Fig. 5A,D,G,J, *H&E stain). We have previously reported the histological analysis of the 8-week HDM model^22^ and observed that the degree of airways inflammation was similar to the 4-week 25 µg model.

### Airway Remodeling

Goblet cell hyperplasia was observed in the airways of the 2- and 4-week HDM mice using PAS stain (Fig. 5C,F,I,L # PAS). However, sub-epithelial collagen deposition (Fig. 5B,E,H,K, blue-green Trichrome Masson) was only observed in the 4-week 25 µg (Fig. 5K, blue-green Trichrome Masson) but not in the 2-week HDM mice (Fig. 5H). Thus, airway remodeling was quantified by measuring the total collagen content in the lungs. The total lung collagen content was increased significantly in both the 4-week 25 μg and 8-week HDM models compared with the Saline controls (Fig. 5M, *p < 0.05, HDM vs. Saline, n = 6/group), with the 8-week HDM mice exhibiting higher collagen content than the 4-week HDM mice (^#^p < 0.05, n = 6/group).

### Inflammatory mediator expression

**Figure 6:**
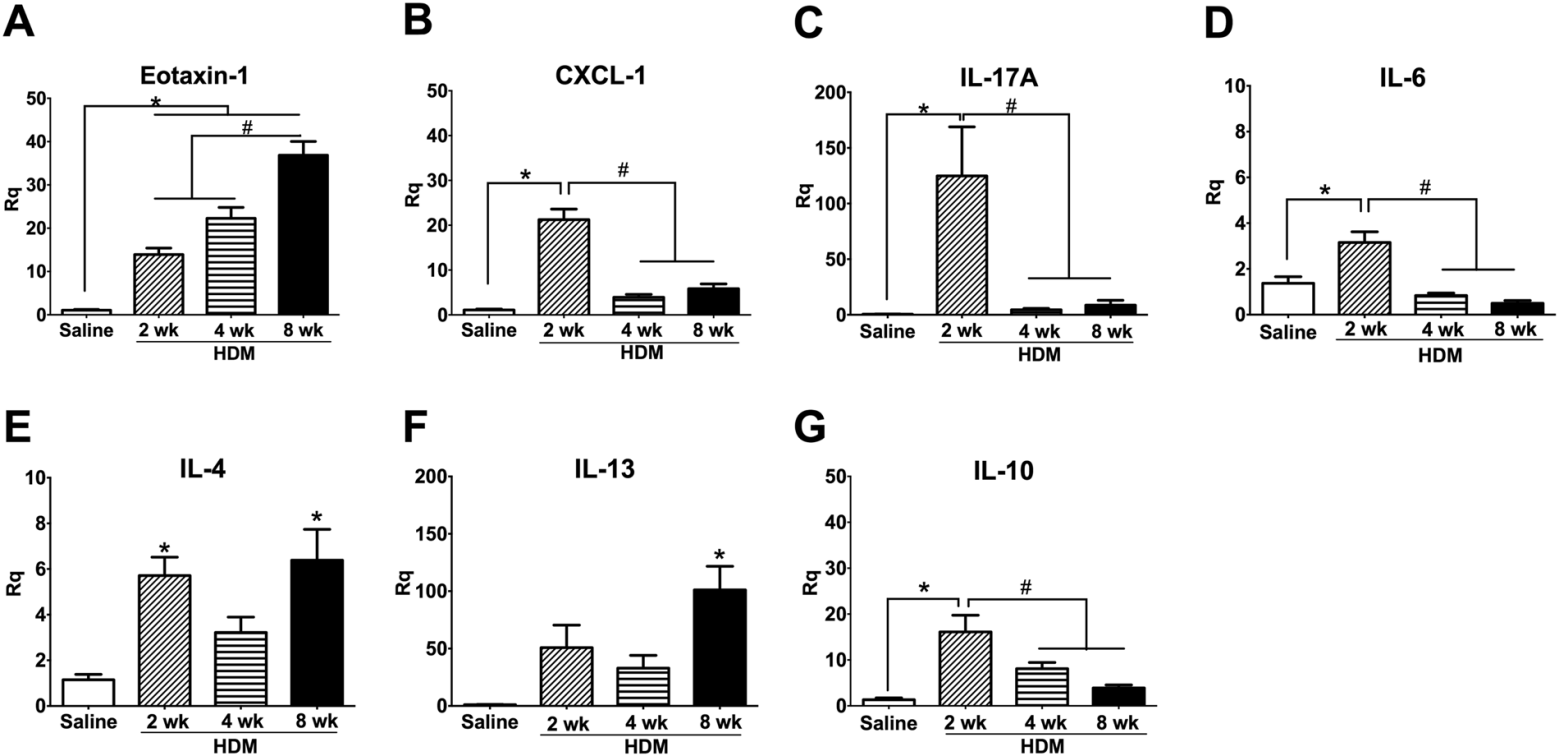
The 3 HDM models exhibit different patterns of inflammatory mediator expression. (**A**) Eotaxin-1 expression increased with increasing duration of HDM sensitization while expression of CXCL-1, IL-17A and IL-6 (**B**–**D**, respectively) was highest at 2 weeks. IL-4 and IL-13 expression (**E**,**F**) showed a biphasic pattern of expression, with the greatest increases seen in the 8-week model. (**G**) IL-10 expression peaked at 2 weeks with progressively lower expression at 4 and 8 weeks (*p < 0.05 vs Saline, ^#^p < 0.05 with HDM groups, n = 6/group).

Evaluation of gene expression in the lungs using qPCR revealed different expression patterns of inflammatory mediators in the HDM models. Of the inflammatory mediators evaluated, eotaxin-1 showed progressively increased expression with the longer durations of HDM exposures (Fig. 6A, *p < 0.05, HDM vs. Saline, n = 6/group). The 2-week HDM mice exhibited prominent up-regulation of CXCL-1, a potent neutrophil chemoattractant, IL-17A and IL-6 expression, that subsequently waned in the 4- and 8-week models (Fig. 6B–D, *p < 0.05, HDM vs Saline, ^#^p < 0.05 vs 2-week HDM, n = 6/group). While IL-4 and IL-13 were increased in all HDM groups compared with Saline controls, the increases were only significantly different in the 2- and 8-week HDM mice (Fig. 6E,F, *p < 0.05 to Saline, n = 6/group). Expression of the anti-inflammatory cytokine, IL-10, was highest in the 2-week HDM mice, and decreased with the duration of HDM exposure (Fig. 6G, *p < 0.05, n = 6/group). We also assessed the expression of other mediators that have been implicated or proposed to play roles in the pathogenesis of asthma (Supplemental Table 1). Of these, while Arg-1 was significantly increased in all HDM-exposed groups compared with Saline controls, Syk and dectin-1 showed significant increases in the 8-week HDM model, with trends toward increases in the 2- and 4-week models (Supplemental Fig. 3).

### Effect of HDM Dose on the 4-Week Model Phenotypes

**Figure 7:**
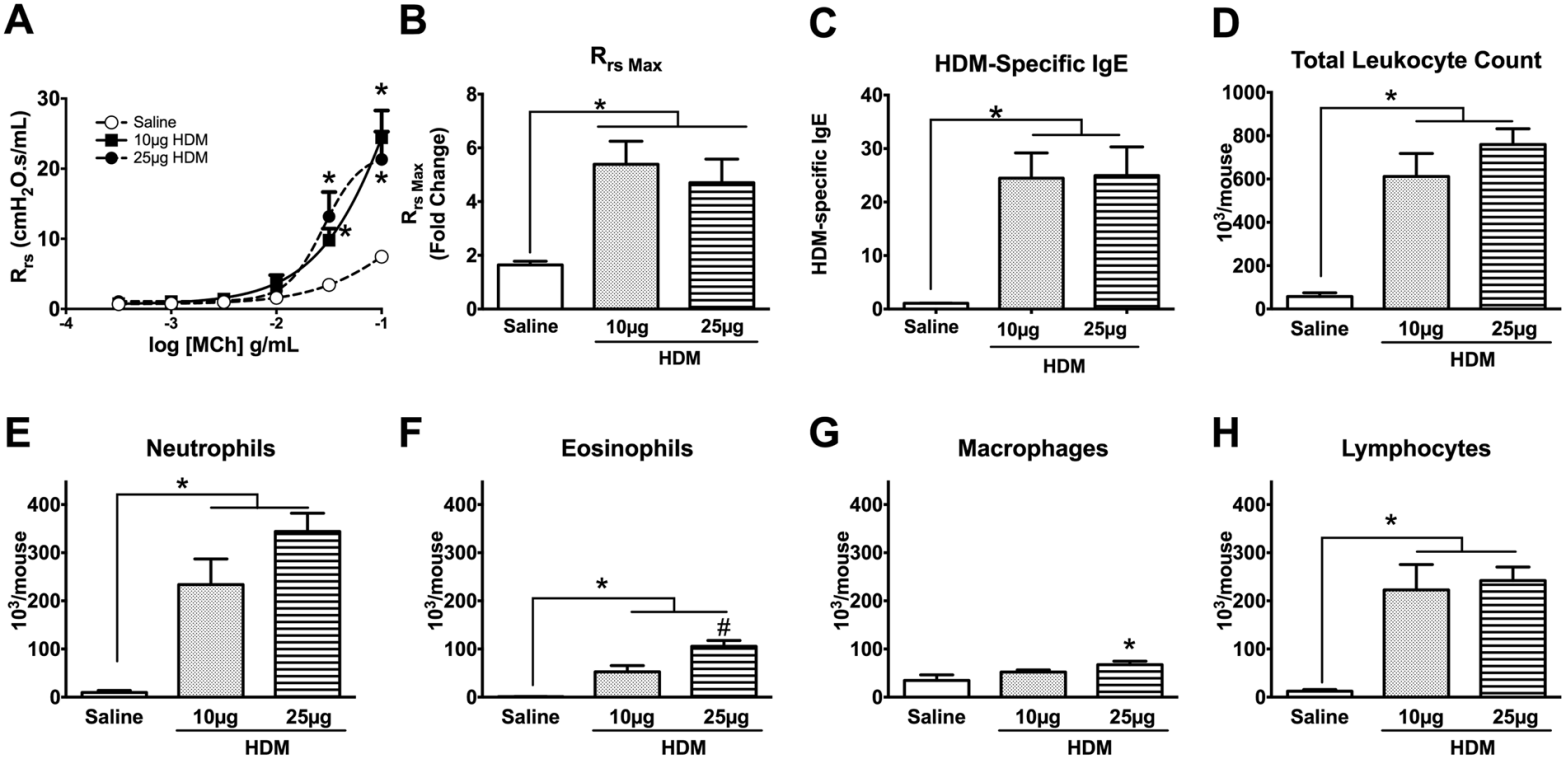
The 10 µg HDM 4-Week Model exhibits a similar degree of AHR and airway inflammation as the 25 µg 4-Week Model. The 10 µg and 25 µg HDM-exposed mice exhibited significant increases in MCh-induced R_rs_ and R_N_ with respect to Saline (**A,B**). This was observed in the MCh-dose responsive curve and maximum R_rs,_ No differences were observed between the 10 µg and the 25 µg HDM groups. Both 10 and 25 µg HDM-exposed developed similar levels of HDM-specific IgE (**C**) and total BAL fluid leukocyte, neutrophil and lymphocyte counts (**D–H**). However, BAL fluid eosinophil counts were significantly higher in the 25 µg compared with the 10 µg HDM mice (**F**). Absolute macrophage counts were significantly increased only in the higher dose HDM model (**G**). *p < 0.05 compared with Saline controls; ^#^p < 0.05 between HDM groups; n = 7/group.

Lastly, we assessed the effect of HDM dose on the resultant phenotype after 4 weeks of allergen exposure by comparing a low (10 µg) to a high (25 µg) HDM dose. We found no difference in R_rs_ or R_rs max_; both dosing protocols significantly increased R_rs_ or R_rs max_ compared to Saline (Fig. 7A,B, *p < 0.05, HDM vs. Saline, n = 7–10/group). Similarly, we observed no significant differences between the two doses with respect to serum HDM-specific IgE levels (Fig. 7C), total BAL fluid leukocyte count (Fig. 7D) or BAL fluid lymphocyte or neutrophil counts (Fig. 7E,H). However, exposure to 25 µg HDM led to significant elevation of eosinophils (Fig. 7F, ^#^p < 0.05) compared with 10 µg HDM.

**Figure 8:**
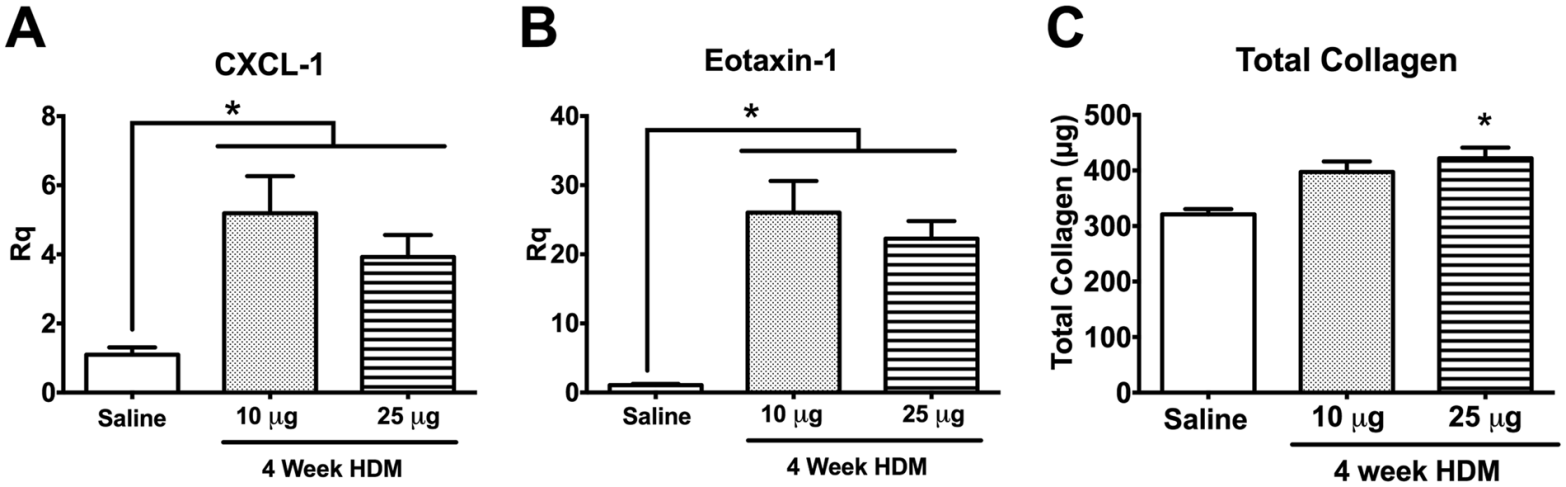
4-week low and high dose HDM exhibit similar upregulation in CXCL-1 and eotaxin-1 expression but increased collagen deposition was only observed with higher HDM dose. Both 4-week HDM doses exhibited similar increase in CXCL-1 and eotaxin-1 expression (**A,B**). However, only the higher dose HDM significantly increased total collagen content relative to Saline (**C**, n = 6–8/group, *p < 0.05).

CXCL-1 and eotaxin-1 were similarly increased with both 4-week HDM doses relative to Saline (Fig. 8A,B). Histological analysis of the lungs exposed to 10 µg HDM for 4 weeks demonstrated increased inflammatory infiltrates, collagen deposition and goblet cell hyperplasia (Supplemental Fig. 4A–C). However, quantitative analysis of the total collagen content revealed that only the higher dose or HDM led to a significant increase (Fig. 8C), suggesting that 10 µg HDM over 4 weeks was insufficient to induce the triad of airways inflammation, AHR and airway remodeling with collagen deposition in the asthma phenotype.

## Discussion

Reversible airflow obstruction, airways inflammation, AHR to contractile agonists (spasmogens, such as methacholine or histamine) and airway remodeling are defining features of asthma^7,10^. Airways inflammation and enhanced MCh-responsiveness are observed in virtually all experimental models of allergic airways inflammation currently used^12,17,20–23,25,26^, with varying degrees depending on the dose, frequency, duration and type of allergen exposure^11,19^. Although goblet cell hyperplasia is observed as early as day 12 in our model (Fig. 5I), and several other reports of 2-week HDM models^12,17^, airway remodeling that also includes subepithelial fibrosis, basement membrane thickening and increased collagen deposition requires a prolonged duration of allergen exposure, typically 5–8 weeks^2,19–22,25^. However, airway remodeling with collagen deposition is a key feature of asthma that has been shown to correlate with asthma severity^28,29^ and to occur in early childhood^6,8,9^. In a study of 44 children with spirometrically-defined asthma, bronchial biopsies revealed significantly increased epithelial loss and basement membrane thickening in children with asthma compared to control subjects and those with non-asthmatic atopic subjects^8^. In a later study, with a different cohort of 55 children that exhibited AHR and wheezing, bronchial biopsies reviewed epithelial loss, basement membrane thickening, angiogenesis, inflammatory cells, IL-4+, and IL-5+ cells typical of asthma in both nonatopic and atopic wheezing children when compared to non-atopic, non-wheezing control subjects^9^. No therapy to-date has shown efficacy in reversing airway remodeling, despite effective treatments for AHR and inflammation. Therefore, studies that are focused on understanding the underlying mechanisms responsible for airway remodeling, and the assessment of therapies that attenuate or reverse collagen deposition could offer the potential to decrease disease severity and improve asthma control. The availability of inexpensive, reliable, and easy-to-establish mouse models that can recapitulate all the key features of asthma (including airway remodeling with collagen deposition) will provide researchers with an improved range of tools to investigate the pathognomonic mechanisms responsible for the development of asthma, and evaluate potential therapies that target airway remodeling. In this paper, we compared the phenotypic features of allergic airways inflammation using HDM, a clinically relevant allergen, and 4 different exposure protocols (Fig. 1). Our 4-week HDM models with daily HDM exposures for 5 days/week exhibited enhanced MCh responsiveness in R_rs_ and R_N_ that were similar to the longer 8-week HDM model.

Airways inflammation, as measured by leukocyte recruitment to the airways, was significantly greater with the longer duration of HDM exposure (Fig. 4) and within the 4-week groups, with the higher dose of HDM (Fig. 7). Similar to the 8-week HDM model^22^, BAL leukocytosis associated with the 4-week HDM models was primarily due to increases in eosinophils and neutrophils. While the absolute BAL eosinophil counts were lowest of the leukocyte sub-populations, the overall fold-increase was the highest, with the 10 µg- and 25 µg HDM 4-week exposure groups exhibiting 6.50- and 10-fold increases, respectively, while the 8-week model exhibited an 8-fold increase relative to Saline controls. In contrast, the increase in neutrophils was only 3-fold in the 4 HDM models compared to Saline controls. Our observations in the 4- and 8-week HDM groups are consistent with previously reported 5-week models^19,20,25^, where robust increases in eosinophils were also accompanied by significant increases in the BAL neutrophil cell counts, with the overall absolute neutrophil counts ~10-fold higher than the absolute eosinophils.

Of the different HDM models evaluated, we observed the highest allergic response (i.e., levels of HDM-specific IgE) in the 4-week model (Fig. 3), with no differences between the lower and higher doses of HDM (Fig. 7C). In our 2-week HDM model, in which mice were exposed to HDM on days 1–5, following by a single challenge on day 11, no increase in HDM-specific IgE was observed on day 12 although we did observe a trend toward a 5.6-fold increase in BAL eosinophils from 1.4% to 7.9% of the total leukocytes; although this increase was not statistically significant. These observations are similar to those of Johnson *et al*., in which consecutive exposure of HDM for 5 days/week failed to induce HDM-IgE expression and eosinophil recruitment after 1 week, but increases in both parameters were observed after 3 weeks^19^. Subsequent studies revealed that 2 full weeks of HDM exposure at 5 consecutive days/week was necessary to elevate HDM-specific IgE levels significantly^17^. Despite the higher dose of HDM used in our 2-week model, differences in the observed HDM-specific IgE levels suggest that ongoing HDM exposures over 2 weeks are essential to inducing the allergic response.

With respect to the allergic airways remodeling in these mice, increased PAS-positive cells were observed as early as 2 weeks after HDM sensitization/exposure (Fig. 5I). However, airway remodeling with collagen deposition was not observed until 4 weeks (Fig. 5K). While Trichrome staining of the airways identified peribronchial deposition of collagen in both the 10 µg and 25 µg 4-week models, quantitative analysis of total lung collagen content indicated that only the higher HDM dosing regimen led to significantly increased collagen deposition compared with Saline controls (Fig. 8C). Collagen deposition also increased with longer durations of HDM exposure, with the highest levels observed in the 8-week HDM model (Fig. 5M). Our findings are similar to previous studies, where major features of airway remodeling, i.e., epithelial thickening, smooth muscle hyperplasia and collagen deposition, were not appreciable after acute exposure to allergens^11,30^, and required sustained allergen exposure of 5 weeks or longer to develop^19,22^. We have previously reported that AHR, airway inflammation and airway remodeling are sustained for at least 2 weeks after cessation of HDM exposure in the 8-week HDM model^22^. We assessed the sustainability of the AHR and airways inflammation in the 4-week 25 µg HDM mice at 6 weeks, and observed that both features reverted to baseline control levels when allergen exposure was ceased at 4 weeks (data not shown), which is consistent with previous studies in which animals recovered after allergen exposure was discontinued^19,25^.

Our survey of inflammatory responses revealed differences in the patterns of leukocyte recruitment to the airways, IgE response and inflammatory mediator expression amongst the different HDM protocols. The neutrophil mediators CXCL-1 and IL-17A were highest at 2 weeks, consistent with the observation that neutrophilic inflammation begins very early, and with observations made in other 2-week HDM-exposure models^17,23^. We also observed significant increases in IL-6 and eotaxin-1 in the 2-week HDM model, although eosinophil recruitment to the airways was not statistically significant, despite a 5.6-fold increase relative to Saline controls. The overall total BAL leukocyte counts were dominated by neutrophils, with absolute cell counts of 2.4 × 10^5^ neutrophils/mouse and 0.06 × 10^5^ eosinophils/mouse; although the relative increase in eosinophil recruitment was higher than that for neutrophils (3.8-fold increase). Our 2-week protocol consisted of daily exposures to 100 µg HDM (*i.n*.) from days 1–5, followed by a single *i.n*. exposure to 100 µg HDM on day 11, and endpoint assessment on day 12. In contrast, in a 2-week model where HDM exposure was sustained continuously for 5 days/week for 2 full weeks, the inflammatory response was eosinophil-dominant, with the absolute BAL eosinophil counts 10-fold higher (in the range of 5 × 10^5^ cells) than the total neutrophil counts (in the range of 0.5 × 10^5^ cells)^17^. In an earlier study of murine allergic airways inflammation^19^, in which the protocol of HDM exposure was conducted using the same dose and frequency as of Piyadasa *et al*.^17^, 1 week of HDM exposure did not result in an allergic response as eosinophil recruitment and induction of HDM-specific IgE were not observed. Differences in the observations may be a result of differences in the dose, frequency and duration of allergen exposure of protocols as well as the specific HDM species used (i.e., *D. pteronyssinus* vs. *D. farinae*).

With increasing durations of HDM exposure, the levels of eotaxin-1 (CCL-11), a potent eosinophil chemoattractant, were also found to be increased. We observed a concomitant progressive increase in the proportion of airway recruitment of eosinophils from 5.6-fold at 2-weeks to 6.5- and 10-fold at 4 weeks (10 µg and 25 µg, respectively) compared with Saline controls. By 8 weeks, the HDM mice exhibited an 8-fold increase in BAL fluid eosinophils compared with Saline controls. Our observations are similar to previous published findings where most models exhibited peak eosinophilia after 3 weeks of allergen exposure, followed by a plateau or non-significant increases in eosinophils thereafter, up to 7 weeks^19,23^. Although the total absolute cell numbers increased with longer durations of HDM exposure, the proportion of neutrophil recruitment to the airways remained stable, 3.0 to 3.8-fold, in all 4 models assessed, suggesting that effect of ongoing allergen exposure was on the eosinophil population. The progressive increase in eotaxin-1 with increasing duration of HDM exposure is supportive of this conclusion.

A hallmark feature of atopic asthma is the elevation of T helper 2 cytokines, i.e., IL-4 and IL-13. We observed a biphasic trend of increases in IL-4 and 1L-13 mRNA expression in the 2- and 8-week models, with the increase in IL-13 only significant after 8 weeks. These observations are similar to previous studies that found increased IL-4 in 2- and 5-week HDM models^17,23^ and IL-13 mRNA levels after 2 weeks of HDM exposure^17^. IL-6 is both a pro-inflammatory cytokine and anti-inflammatory cytokine that has been implicated in the pathogenesis of asthma as well as a biomarker of asthma exacerbations^31^, while IL-10 is an anti-inflammatory cytokine that has been postulated to regulate resolution of airways inflammation^32^. In our models, expression of both mediators peaked at 2 weeks, with progressive decreases at 4 and 8 weeks of HDM exposure, which corresponds temporally with the progressive increase in airways inflammation and airway remodeling.

In this study, we found that while all 4 different models of HDM-induced allergic airways inflammation exhibited AHR, they exhibited different phenotypic features of airways inflammation, systemic allergic response and airways remodeling. These differences are likely related to the variations in the frequency, dosing and duration of allergen exposure. Of the models evaluated, our 2-week model showed an early inflammatory response dominated by neutrophil inflammation and upregulation of related mediators, i.e., CXCL-1 and IL-17A. Although we detected significant increases in eotaxin-1 gene expression, no statistically significant increases in eosinophil recruitment or serum HDM-specific IgE were observed. It should be noted that our acute HDM protocol is different from previously published 2-week models in which HDM exposure was conducted continuously for 2 full weeks for 5 days/week, and where AHR was accompanied by airways inflammation associated with an allergic response, manifested by prominent eosinophilic infiltration and elevated HDM- specific IgE^17,30^. In contrast, our 2-week model consisted of HDM exposure on days 1–5, following by a single exposure on day 11 and outcome metric assessment on day 12. We propose that our 2-week (12-day) model is a useful tool for the assessment of general airway inflammation, where the allergic phenotype (i.e., allergen-specific IgE accumulation) is not the focus of interest, whereas the 2-week continuous allergen exposure models are better suited for the assessment of allergic airways inflammation. Although our acute model, like the other 2 week models^17^ exhibit goblet cell hyperplasia, other features of airway remodeling remain absent. These include the prominent features of the asthmatic airway in humans, namely sub-epithelial fibrosis, basement membrane thickening, airway smooth muscle hyperplasia and collagen deposition. As a result, 2-week models of allergic airways inflammation are useful for pilot, preclinical, or proof-of-concept projects in evaluating potential therapeutic interventions or in mechanistic studies focused on the early inflammatory pathways, prior to investing significant resources and time in developing chronic allergic airways inflammation models that manifest airway remodeling with collagen deposition.

To the best of our knowledge, the 4-week HDM model described in the current manuscript is the shortest protocol that leads to development of AHR, airway inflammation and airway remodeling with collagen deposition. While both the 10 µg and 25 µg models manifest similar degrees of AHR and airways inflammation, and subjective evidence of collagen deposition, quantitative analysis of the total lung collagen content showed that the lower HDM dose is insufficient for development of a model of asthma that produces significant and quantifiable airways remodeling.

The many different models of allergic airways inflammation with well-characterized phenotypic feature offer a choice of tools for researchers to exploit, when designing studies to evaluate airway inflammation, allergic inflammation and asthma. This breadth of choice facilitates the selection of specific models with careful consideration to the specific features in interest. Of the 4 HDM models described in the current manuscript, we propose that both the 4- and 8-week 25 µg HDM models are the optimal choice for experimental studies of asthma, as the presence of AHR, airways inflammation and airway remodeling with collagen deposition are more representative of the clinical observations in asthmatic patients. While there are differences with respect to the degree and type of leukocyte recruitment, airway inflammation and airway remodeling between the 4- and 8-week models presented herein, and the previously published chronic models of 5–8 week duration^19–25^, the 4-week 25 µg HDM model offers advantages in the economy of time and resources, while modeling the main characteristics of asthma.

## Electronic supplementary material

Supplemental Information
