# Supplementary material for: A 4-Week Model of House Dust Mite (HDM) Induced Allergic Airways Inflammation with Airway Remodeling
Source: Sci Rep. 2018 May 2;8:6925. doi: 10.1038/s41598-018-24574-x (PMC5932037; doi:10.1038/s41598-018-24574-x)
Supplement: Supplementary file 1 — Supplemental Information [file 41598_2018_24574_MOESM1_ESM.pdf]

A 4-Week Model of House Dust Mite (HDM) Induced Allergic Airways Inflammation with Airway Remodeling

LN Woo[1*], WY Guo[1*], X Wang[1], A. Young[1], S. Salehi[1], A. Hin[1], Y. Zhang[1], JA Scott[2], CW Chow[1,2#]

[1]Division of Respirology and Multi-Organ Transplant Programme, University Health Network, Faculty of Medicine, [2]Division of Occupational Health, Dalla Lana School of Public Health, University of Toronto

* LN Woo and WY Guo contributed equally to the manuscript.

Running title: A 4-week chronic mouse model of HDM-induced airways remodeling

Keywords: Allergy, Asthma, House Dust Mite, Mouse, Airways Inflammation, Airway Hyper-responsiveness, methacholine, Inflammation, Remodeling

#Corresponding author and contact information:

Dr. Chung-Wai Chow
585 University Avenue, 11 PMB 130
Toronto, ON
Canada M5G 2N2
cw.chow@utoronto.ca

Supplemental Materials

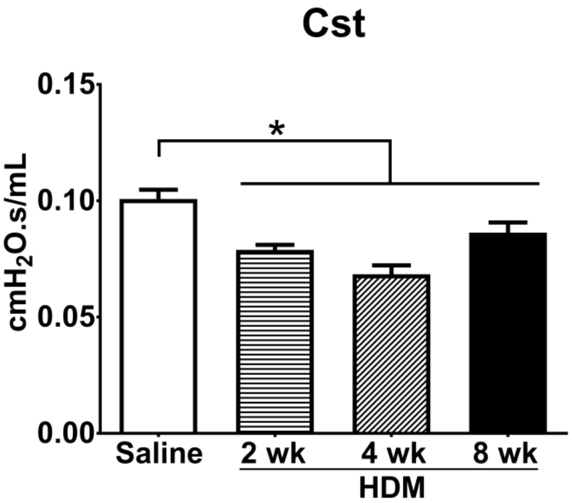

Supplementary Figure 1: Quasistatic Lung Compliance (C_{st}) is Reduced by all HDM (*D. pteronyssinus*) exposures. C_{st} is reduced in all HDM models compared to Saline controls (*p<0.05).

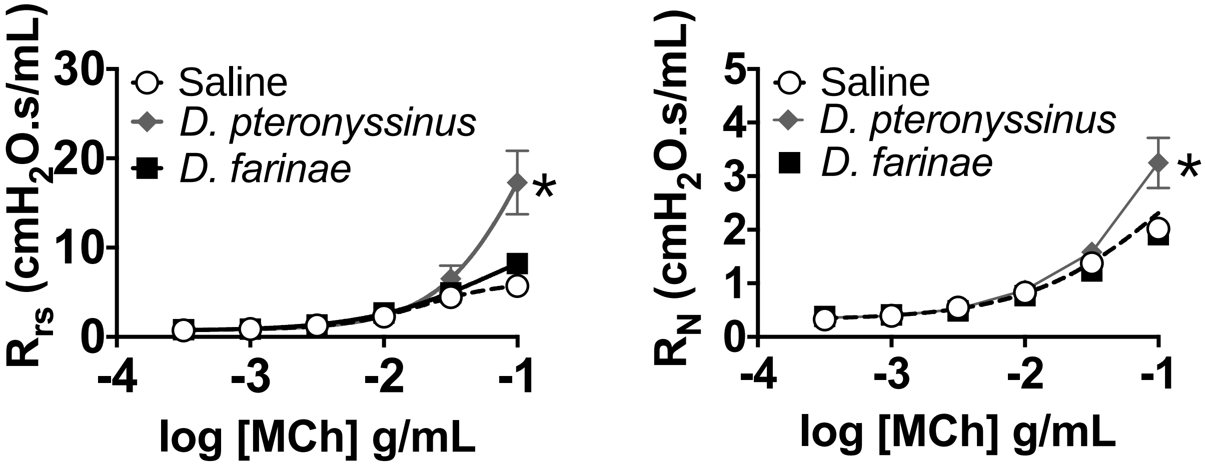

Supplementary Figure 2: Different species of HDM induced variable AHR. Two weeks of HDM exposure led to the development of AHR (R_{rs} and R_N) to methacholine with *D. pteronyssinus*, but not *D. farinae* (*p<0.05).

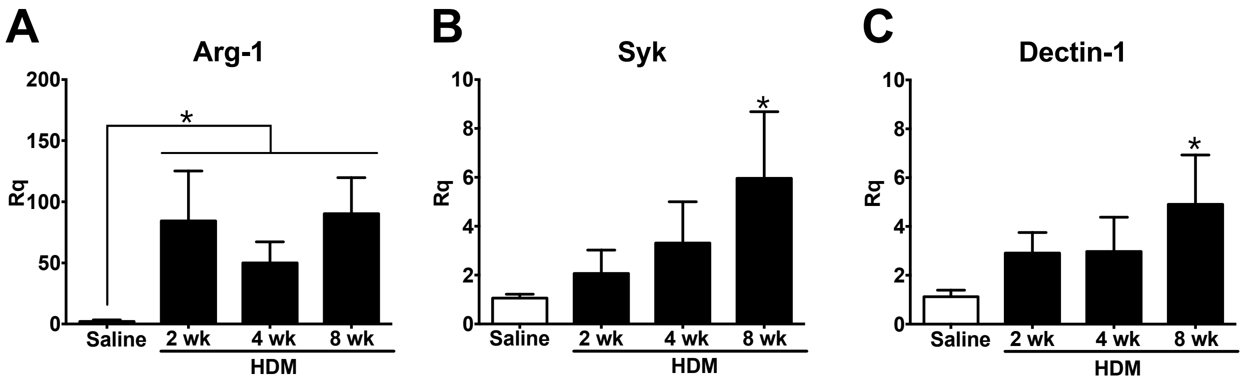

Supplementary Figure 3: Expression of Syk, Dectin-1 and Arg-1 is upregulated with HDM exposure. While Arg-1 expression was upregulated in all HDM models with respect to Saline (**A**), Syk and dectin-1 expression were significantly increased in the 8-week HDM model compared with Saline, with increased trends in the 2- and 4-week models (**B,C**). n=6/group, *p<0.05.

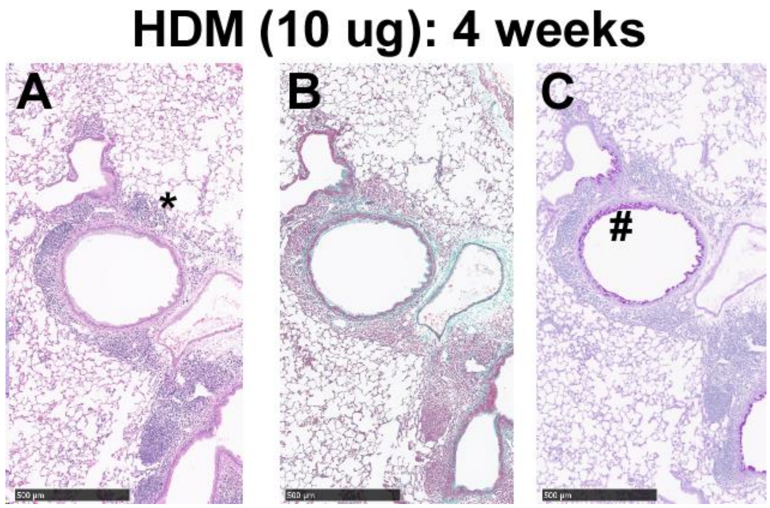

Supplementary Figure 4: Histological Analysis of the 4-week 10 µg HDM-exposed lungs show similar degree of inflammatory infiltrates, collagen deposition and goblet hyperplasia to the 4-week 25 µg HDM exposed lugns. Inflammatory infiltrates (A, * H&E stain), collagen deposition (B) and goblet hyperplasia (C, # PAS stain) were observed in both the 4-week 10 µg and 4-week 25 µg HDM-exposed animals.

Supplemental Table 1. TaqMan® Gene expression primers.

Gene	Assay ID
peptidylprolyl isomerase A pseudogene 8 (Ppia-ps8)	Mm02342429_g1
chemokine (C-C motif) ligand 11 (Eotaxin, CcL-11)	Mm00441238_m1
chemokine (C-X-C motif) ligand 1 (CXCL-1)	Mm04207460_m1
interleukin 4 (IL-4)	Mm00445259_m1
interleukin 6 (IL-6)	Mm00446190_m1
interleukin 10 (IL-10)	Mm01288386_m1
interleukin 13 (IL-13)	Mm00434204_m1
interleukin 17A (IL-17A)	Mm00439618_m1
spleen tyrosine kinase (Syk)	Mm01333032_m1
C-type lectin domain family 7, member a (CLEC7A, Dectin-1)	Mm01183349_m1
arginase, liver (Arg-1)	Mm00475988_m1
